# Design of a new *Z*-test for the uncertainty of Covid-19 events under Neutrosophic statistics

**DOI:** 10.1186/s12874-022-01593-x

**Published:** 2022-04-06

**Authors:** Muhammad Aslam

**Affiliations:** Department of Statistics, Faculty of Science, King Abdulaziz University, Jeddah, 21551 Saudi Arabia

**Keywords:** Uncertainty, Classical statistics, Z test, Simulation, Data

## Abstract

**Background:**

The existing Z-test for uncertainty events does not give information about the measure of indeterminacy/uncertainty associated with the test.

**Methods:**

This paper introduces the Z-test for uncertainty events under neutrosophic statistics. The test statistic of the existing test is modified under the philosophy of the Neutrosophy. The testing process is introduced and applied to the Covid-19 data.

**Results:**

Based on the information, the proposed test is interpreted as the probability that there is no reduction in uncertainty of Covid-19 is accepted with a probability of 0.95, committing a type-I error is 0.05 with the measure of an indeterminacy 0.10. Based on the analysis, it is concluded that the proposed test is informative than the existing test. The proposed test is also better than the Z-test for uncertainty under fuzzy-logic as the test using fuzz-logic gives the value of the statistic from 2.20 to 2.42 without any information about the measure of indeterminacy. The test under interval statistic only considers the values within the interval rather than the crisp value.

**Conclusions:**

From the Covid-19 data analysis, it is found that the proposed Z-test for uncertainty events under the neutrosophic statistics is efficient than the existing tests under classical statistics, fuzzy approach, and interval statistics in terms of information, flexibility, power of the test, and adequacy.

## Background

The Z-test is playing an important role in analyzing the data. The main aim of the Z-test is to test the mean of the unknown population in decision-making. The Z-test for uncertainty events is applied to test the reduction in the uncertainty of past events. This type of test is applied to test the null hypothesis that there is no reduction in uncertainty against the alternative hypothesis that there is a significant reduction in uncertainty of past events. The Z-test for uncertainty events uses the information of the past events for testing the reduction of uncertainty [1]. discussed the performance of the statistical test under uncertainty [2]. discussed the design of the Z-test for uncertainty events [3]. worked on the test in the presence of uncertainty [4]. worked on the modification of non-parametric test. The applications of [5], [6], [7] and [8].

[9] mentioned that “statistical data are frequently not precise numbers but more or less non-precise also called fuzzy. Measurements of continuous variables are always fuzzy to a certain degree”. In such cases, the existing Z-tests cannot be applied for the testing of the mean of population or reduction in uncertainty. Therefore, the existing Z-tests are modified under the fuzzy-logic to deal with uncertain, fuzzy, and vague data [10]., [11], [12], [13], [14], [15], [16], [17], [18], [19] worked on the various statistical tests using the fuzzy-logic.

Nowadays, neutrosophic logic attracts researchers due to its many applications in a variety of fields. The neutrosophic logic counters the measure of indeterminacy that is considered by the fuzzy logic, see [20] [21]. proved that neutrosophic logic is efficient than interval-based analysis. More applications of neutrosophic logic can be seen in [22], [23], [24] and [25] [26]. applied the neutrosophic statistics to deal with uncertain data [27]. and [28] presented neutrosophic statistical methods to analyze the data. Some applications of neutrosophic tests can be seen in [29], [30] and [31].

The existing Z-test for uncertainty events under classical statistics does not consider the measure of indeterminacy when testing the reduction in events. By exploring the literature and according to the best of our knowledge, there is no work on Z-test for uncertainty events under neutrosophic statistics. In this paper, the medication of Z-test for uncertainty events under neutrosophic statistics will be introduced. The application of the proposed test will be given using the Covid-19 data. It is expected that the proposed Z-test for uncertainty events under neutrosophic statistics will be more efficient than the existing tests in terms of the power of the test, information, and adequacy.

## Methods

The existing Z-test for uncertainty events can be applied only when the probability of events is known. The existing test does not evaluate the effect of the measure of indeterminacy/uncertainty in the reduction of uncertainty of past events. We now introduce the modification of the Z-test for uncertainty events under neutrosophic statistics. With the aim that the proposed test will be more effective than the existing Z-test for uncertainty events under classical statistics. Let ${A}_N={A}_L+{A}_U{I}_{A_N};{I}_{A_N}\epsilon \left[{I}_{A_L},{I}_{A_U}\right]$ and ${B}_N={B}_L+{B}_U{I}_{B_N};{I}_{B_N}\epsilon \left[{I}_{B_L},{I}_{B_U}\right]$ be two neutrosophic events, where lower values *A*_*L*_, *B*_*L*_ denote the determinate part of the events, upper values ${A}_U{I}_{A_N}$, ${B}_U{I}_{B_N}$ be the indeterminate part, and ${I}_{A_N}\epsilon \left[{I}_{A_L},{I}_{A_U}\right]$, ${I}_{B_N}\epsilon \left[{I}_{B_L},{I}_{B_U}\right]$ be the measure of indeterminacy associated with these events. Note here that the events *A*_*N*_*ϵ*[*A*_*L*_, *A*_*U*_] and *B*_*N*_*ϵ*[*B*_*L*_, *B*_*U*_] reduces to events under classical statistics (determinate parts) proposed by [2] if ${I}_{A_L}={I}_{B_L}$ =0. Suppose *n*_*N*_ = *n*_*L*_ + *n*_*U*_*I*_*N*_; *I*_*N*_*ϵ*[*I*_*L*_, *I*_*U*_] be a neutrosophic random sample where *n*_*L*_ is the lower (determinate) sample size and *n*_*U*_*I*_*N*_ be the indeterminate part and *I*_*N*_*ϵ*[*I*_*L*_, *I*_*U*_] be the measure of uncertainty in selecting the sample size. The neutrosophic random sample reduces to random sample if no uncertainty is found in the sample size. The methodology of the proposed Z-test for uncertainty events is explained as follows.

Suppose that the probability that an event *A*_*N*_*ϵ*[*A*_*L*_, *A*_*U*_] occurs (probability of truth) is *P*(*A*_*N*_)*ϵ*[*P*(*A*_*L*_), *P*(*A*_*U*_)], the probability that an event *A*_*N*_*ϵ*[*A*_*L*_, *A*_*U*_] does not occur (probability of false) is $P\left({A}_N^c\right)\epsilon \left[P\left({A}_L^c\right),P\left({A}_U^c\right)\right]$, the probability that an event *B*_*N*_*ϵ*[*B*_*L*_, *B*_*U*_] occurs (probability of truth) is *P*(*B*_*N*_)*ϵ*[*P*(*B*_*L*_), *P*(*B*_*U*_)], the probability that an event *B*_*N*_*ϵ*[*B*_*L*_, *B*_*U*_] does not occur (probability of false) is $P\left({B}_N^c\right)\epsilon \left[P\left({B}_L^c\right),P\left({B}_U^c\right)\right]$. It is important to note that sequential analysis is done to reduce the uncertainty by using past events information. The purpose of the proposed test is whether the reduction of uncertainty is significant or not. Let *Z*_*N*_*ϵ*[*Z*_*L*_, *Z*_*U*_] be neutrosophic test statistic, where *Z*_*L*_ and *Z*_*U*_ are the lower and upper values of statistic, respectively and defined by.

1 $${Z}_N\epsilon \left\{\frac{P\left({B}_{+ kL}|{A}_L\right)-P\left({B}_L\right)}{\sqrt{\frac{P\left({B}_L\right)\left[1-P\left({B}_L\right)\right]\left[1-P\left({A}_L\right)\right]}{\left({n}_L-{k}_L\right)P\left({A}_L\right)}}},\frac{P\left({B}_{+ kU}|{A}_U\right)-P\left({B}_U\right)}{\sqrt{\frac{P\left({B}_U\right)\left[1-P\left({B}_U\right)\right]\left[1-P\left({A}_U\right)\right]}{\left({n}_U-{k}_U\right)P\left({A}_U\right)}}}\right\}$$

Note that *P*(*B*_+*kN*_| *A*_*N*_) = *P*(*B*_*N*_| *A*_*N*_) at lag *k*_*N*_, where *P*(*B*_*N*_| *A*_*N*_)*ϵ*[*P*(*B*_*L*_| *A*_*L*_), *P*(*B*_*U*_| *A*_*U*_)] denotes the conditional probability. It means that the probability of event *P*(*B*_*N*_)*ϵ*[*P*(*B*_*L*_), *P*(*B*_*U*_)] will be calculated when the event *A*_*N*_*ϵ*[*A*_*L*_, *A*_*U*_] has occurred.

The neutrosophic form of the proposed test statistic, say *Z*_*N*_*ϵ*[*Z*_*L*_, *Z*_*U*_] is defined by.

2 $${Z}_N=\frac{P\left({B}_{+ kL}|{A}_L\right)-P\left({B}_L\right)}{\sqrt{\frac{P\left({B}_L\right)\left[1-P\left({B}_L\right)\right]\left[1-P\left({A}_L\right)\right]}{\left({n}_L-{k}_L\right)P\left({A}_L\right)}}}+\frac{P\left({B}_{+ kU}|{A}_U\right)-P\left({B}_U\right)}{\sqrt{\frac{P\left({B}_U\right)\left[1-P\left({B}_U\right)\right]\left[1-P\left({A}_U\right)\right]}{\left({n}_U-{k}_U\right)P\left({A}_U\right)}}}{I}_{ZN};{I}_{ZN}\epsilon \left[{I}_{ZL},{I}_{ZU}\right]$$

The alternative form of Eq. (2) can be written as.

3 $${Z}_N=\left(1+{I}_{ZN}\right)\frac{P\left({B}_{+ kN}|{A}_N\right)-P\left({B}_N\right)}{\sqrt{\frac{P\left({B}_N\right)\left[1-P\left({B}_N\right)\right]\left[1-P\left({A}_N\right)\right]}{\left({n}_N-{k}_N\right)P\left({A}_N\right)}}};{I}_{ZN}\epsilon \left[{I}_{ZL},{I}_{ZU}\right]$$

The proposed test *Z*_*N*_*ϵ*[*Z*_*L*_, *Z*_*U*_] is the extension of several existing tests. The proposed test reduces to the existing Z test under classical statistics when *I*_*ZN*_ =0. The proposed test is also an extension of the Z test under fuzzy approach and interval statistics.

The proposed test will be implemented as follows.

**Step-1:** state the null hypothesis *H*_0_: there is no reduction in uncertainty vs. the alternative hypothesis *H*_1_: there is a significant reduction in uncertainty.

**Step-2:** Calculate the statistic *Z*_*N*_*ϵ*[*Z*_*L*_, *Z*_*U*._]

**Step-3:** Specify the level of significance *α* and select the critical value from [2].

**Step-4:** Do not accept the null hypothesis if the value of *Z*_*N*_*ϵ*[*Z*_*L*_, *Z*_*U*_] is larger than the critical value.

## Results

The application of the proposed test is given in the medical field. The decision-makers are interested to test the reduction in uncertainty of Covid-19 when the measure of indeterminacy/uncertainty is *I*_*ZN*_*ϵ*[0,0.10]. The decision-makers are interested to test that the reduction in death due to Covid-19 (event *A*_*N*_) with the increase in Covid-19 vaccines (event *B*_*N*_). By following [2], the sequence in which both events occur is given as

$${A}_N\ {A}_N,{B}_N\ {A}_N,{B}_N\ {A}_N,{B}_N\ {B}_N,{A}_N\ {B}_N,{A}_N\ {B}_N$$

where *n*_*N*_*ϵ*[12, 12], *k*_*N*_*ϵ*[1, 1], *P*(*A*_*N*_) = 6/12 = 0.5 and *P*(*B*_*N*_) = 6/12 = 0.5.

Note here that event *A*_*N*_ occurs 6 times and that of these 6 times *B*_*N*_ occurs immediately after *A*_*N*_ five times. Given that *A*_*N*_ has occurred, we get

(*B*_+*kN*_| *A*_*N*_) = *P*(*B*_*N*_| *A*_*N*_) = 5/6 = 0.83 at lag 1. The value of *Z*_*N*_*ϵ*[*Z*_*L*_, *Z*_*U*_] is calculated as

${Z}_N=\left(1+0.1\right)\frac{0.83-0.50}{\sqrt{\frac{0.50\left[1-0.50\right]\left[1-0.50\right]}{\left(12-1\right)0.50}}}=2.42;{I}_{ZN}\epsilon \left[\mathrm{0,0.1}\right]$. From [2], the critical value is 1.96.

The proposed test for the example will be implemented as follows

**Step-1:** state the null hypothesis *H*_0_: there is no reduction in uncertainty of Covid-19 vs. the alternative hypothesis *H*_1_: there is a significant reduction in uncertainty of Covid-19.

**Step-2:** the value of the statistic is 2.42.

**Step-3:** Specify the level of significance *α* = 0.05 and select the critical value from [2] which is 1.96.

**Step-4:** Do not accept the null hypothesis as the value of *Z*_*N*_ is larger than the critical value.

From the analysis, it can be seen that the calculated value of *Z*_*N*_*ϵ*[*Z*_*L*_, *Z*_*U*_] is larger than the critical value of 1.96. Therefore, the null hypothesis *H*_0_: there is no reduction in uncertainty of Covid-19 will be rejected in favor of *H*_1_: there is a significant reduction in uncertainty of Covid-19. Based on the study, it is concluded that there is a significant reduction in the uncertainty of Covid-19.

### Simulation study

In this section, a simulation study is performed to see the effect of the measure of indeterminacy on statistic *Z*_*N*_*ϵ*[*Z*_*L*_, *Z*_*U*_]. For this purpose, a neutrosophic form of *Z*_*N*_*ϵ*[*Z*_*L*_, *Z*_*U*_] obtained from the real data will be used. The neutrosophic form of *Z*_*N*_*ϵ*[*Z*_*L*_, *Z*_*U*_] is given as

$${Z}_N=\left(1+{I}_{ZN}\right)\frac{0.83-0.50}{\sqrt{\frac{0.50\left[1-0.50\right]\left[1-0.50\right]}{\left(12-1\right)0.50}}};{I}_{ZN}\epsilon \left[{I}_{ZL},{I}_{ZU}\right]$$

To analyze the effect on *H*_0_, the various values of *I*_*ZN*_*ϵ*[*I*_*ZL*_, *I*_*ZU*_] are considered. The computed values of *Z*_*N*_*ϵ*[*Z*_*L*_, *Z*_*U*_] along with the decision on *H*_0_ are reported in Table 1. For this study *α* = 0.05 and the critical value is 1.96. The null hypothesis *H*_0_ will be accepted if the calculated value of *Z*_*N*_ is less than 1.96. From Table 1, it can be seen that as the values of *I*_*ZN*_*ϵ*[*I*_*ZL*_, *I*_*ZU*_] increases from 0.01 to 2, the values of *Z*_*N*_*ϵ*[*Z*_*L*_, *Z*_*U*_] increases. Although, a decision about *H*_0_ remains the same at all values of measure of indeterminacy *I*_*ZN*_*ϵ*[*I*_*ZL*_, *I*_*ZU*_] but the difference between *Z*_*N*_*ϵ*[*Z*_*L*_, *Z*_*U*_] and the critical value of 1.96 increases as *I*_*ZU*_ increases. From the study, it can be concluded that the measure of indeterminacy *I*_*ZN*_*ϵ*[*I*_*ZL*_, *I*_*ZU*_] affects the values of *Z*_*N*_*ϵ*[*Z*_*L*_, *Z*_*U*_].

**Table 1:** The effect on *Z*_*N*_*ϵ*[*Z*_*L*_, *Z*_*U*_] for various values of *I*_*ZN*_*ϵ*[*I*_*ZL*_, *I*_*ZU*_]

*I*_*ZN*_*ϵ*[*I*_*ZL*_, *I*_*ZU*_]	*Z*_*N*_*ϵ*[*Z*_*L*_, *Z*_*U*_]	Decision about *H*_0_	*I*_*ZN*_*ϵ*[*I*_*ZL*_, *I*_*ZU*_]	*Z*_*N*_*ϵ*[*Z*_*L*_, *Z*_*U*_]	Decision about *H*_0_
[0,0]	[2.20,2.20]	Reject *H*_0_	[0,0.15]	[2.20,2.53]	Reject *H*_0_
[0,0.01]	[2.20,2.22]	Reject *H*_0_	[0,0.16]	[2.20,2.55]	Reject *H*_0_
[0,0.02]	[2.20,2.24]	Reject *H*_0_	[0,0.17]	[2.20,2.57]	Reject *H*_0_
[0,0.03]	[2.20,2.26]	Reject *H*_0_	[0,0.18]	[2.20,2.59]	Reject *H*_0_
[0,0.04]	[2.20,2.28]	Reject *H*_0_	[0,0.19]	[2.20,2.61]	Reject *H*_0_
[0,0.05]	[2.20,2.31]	Reject *H*_0_	[0,0.2]	[2.20,2.64]	Reject *H*_0_
[0,0.06]	[2.20,2.33]	Reject *H*_0_	[0,0.3]	[2.20,2.86]	Reject *H*_0_
[0,0.07]	[2.20,2.35]	Reject *H*_0_	[0,0.4]	[2.20,3.08]	Reject *H*_0_
[0,0.08]	[2.20,2.37]	Reject *H*_0_	[0,0.5]	[2.20,3.30]	Reject *H*_0_
[0,0.09]	[2.20,2.39]	Reject *H*_0_	[0,0.6]	[2.20,3.52]	Reject *H*_0_
[0,0.10]	[2.20,2.42]	Reject *H*_0_	[0,0.7]	[2.20,3.74]	Reject *H*_0_
[0,0.11]	[2.20,2.44]	Reject *H*_0_	[0,0.8]	[2.20,3.96]	Reject *H*_0_
[0,0.12]	[2.20,2.46]	Reject *H*_0_	[0,0.9]	[2.20,4.18]	Reject *H*_0_
[0,0.13]	[2.20,2.48]	Reject *H*_0_	[0,1]	[2.20,4.40]	Reject *H*_0_
[0,0.14]	[2.20,2.50]	Reject *H*_0_	[0,2]	[2.20,6.60]	Reject *H*_0_

### Comparative studies

As mentioned earlier, the proposed Z-test for uncertainty events is an extension of several tests. In this section, a comparative study is presented in terms of measure of indeterminacy, flexibility and information. We will compare the efficiency of the proposed Z-test for uncertainty with the proposed Z-test for uncertainty under classical statistics, proposed Z-test for uncertainty under fuzzy logic and proposed Z-test for uncertainty under interval statistics. The neutrosophic form of the proposed statistic *Z*_*N*_*ϵ*[*Z*_*L*_, *Z*_*U*_] is expressed as *Z*_*N*_ = 2.20 + 2.20*I*_*ZN*_; *I*_*ZN*_*ϵ*[0,0.1]. Note that the first 2.20 presents the existing Z-test for uncertainty under classical statistics, the second part 2.20*I*_*ZN*_ is an indeterminate part and 0.1 is a measure of indeterminacy associated with the test. From the neutrosophic form, it can be seen that the proposed test is flexible as it gives the values of *Z*_*N*_*ϵ*[*Z*_*L*_, *Z*_*U*_] in an interval from 2.20 to 2.42 when *I*_*ZU*_ =0. On the other hand, the existing test gives the value of 2.20. In addition, the proposed test uses information about the measure of indeterminacy that the existing test does not consider. Based on the information, the proposed test is interpreted as the probability that *H*_0_: there is no reduction in uncertainty of Covid-19 is accepted with a probability of 0.95, committing a type-I error is 0.05 with the measure of an indeterminacy 0.10. Based on the analysis, it is concluded that the proposed test is informative than the existing test. The proposed test is also better than the Z-test for uncertainty under fuzzy-logic as the test using fuzz-logic gives the value of the statistic from 2.20 to 2.42 without any information about the measure of indeterminacy. The test under interval statistic only considers the values within the interval rather than the crisp value. On the other hand, the analysis based on neutrosophic considers any type of set. Based on the analysis, it is concluded that the proposed Z-test is efficient than the existing tests in terms of information, flexibility, and indeterminacy.

### Comparison using power of the test

**Fig. 1:**
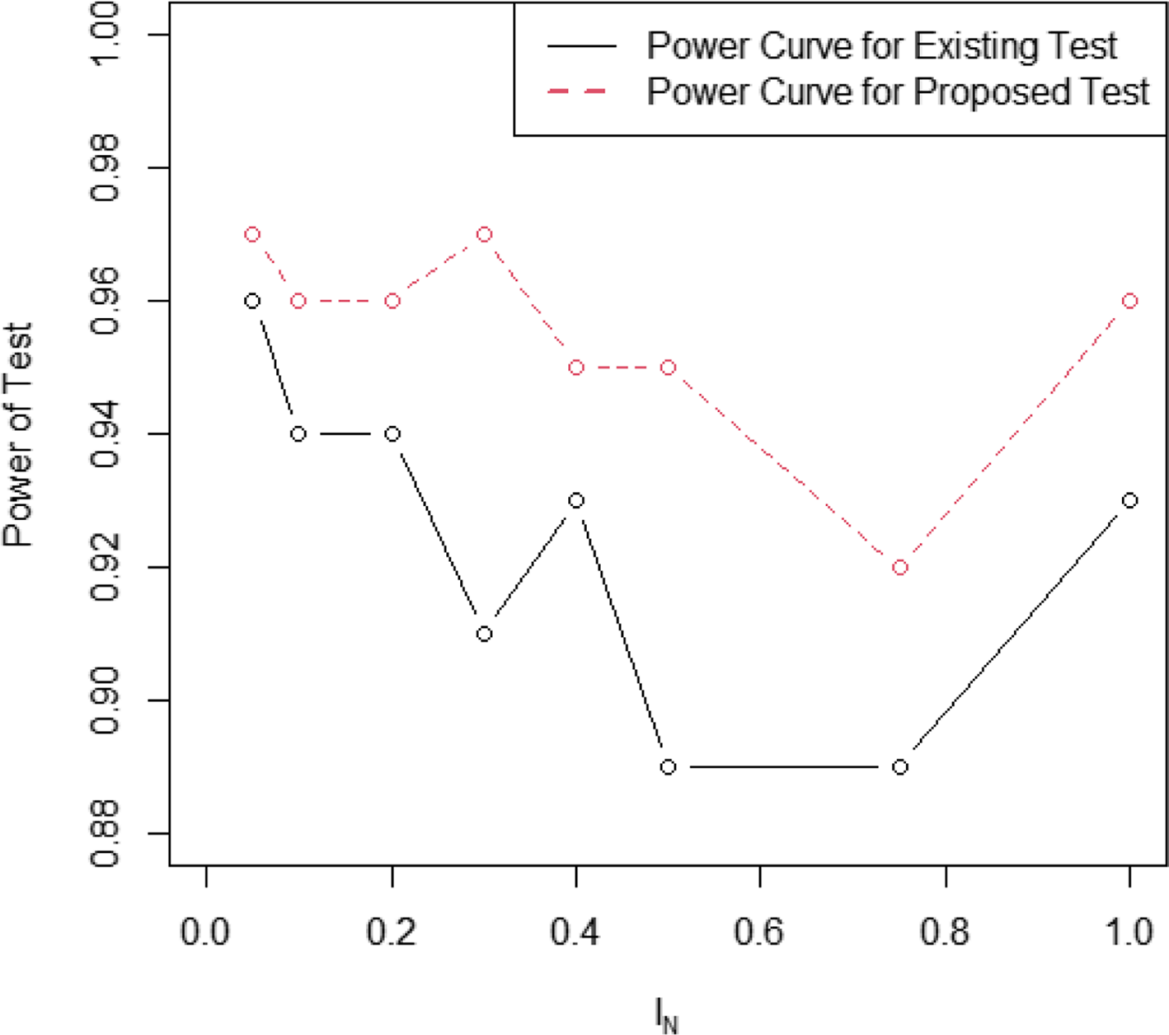
The power curves of the two tests

In this section, the efficiency of the proposed test is compared with the existing test in terms of the power of the test. The power of the test is defined as the probability of rejecting *H*_0_ when it is false and it is denoted by *β*. As mentioned earlier, the probability of rejecting *H*_0_ when it is true is known as a type-I error is denoted by *α*. The values of *Z*_*N*_*ϵ*[*Z*_*L*_, *Z*_*U*_] are simulated using the classical standard normal distribution and neutrosophic standard normal distribution. During the simulation 100 values of *Z*_*N*_*ϵ*[*Z*_*L*_, *Z*_*U*_] are generated from a classical standard normal distribution and neutrosophic standard normal distribution with mean ${\mu}_N={\mu}_L+{\mu}_U{I}_{\mu_N};{I}_{\mu_N}\epsilon \left[{I}_{\mu_L},{I}_{\mu_U}\right]$, where *μ*_*L*_ = 0 presents the mean of classical standard normal distribution, ${\mu}_U{I}_{\mu_N}$ denote the indeterminate value and ${I}_{\mu_N}\epsilon \left[{I}_{\mu_L},{I}_{\mu_U}\right]$ is a measure of indeterminacy. Note that when ${I}_{\mu_L}$ =0, *μ*_*N*_ reduces to *μ*_*L*_. The values of *Z*_*N*_*ϵ*[*Z*_*L*_, *Z*_*U*_] are compared with the tabulated value at *α* =0.05. The values of the power of the test for the existing test and for the proposed test for various values of ${I}_{\mu_U}$ are shown in Table 2. From Table 2, it is clear that the existing test under classical statistics provides smaller values of the power of the test as compared to the proposed test at all values of ${I}_{\mu_U}$. For example, when ${I}_{\mu_U}$ =0.1, the power of the test provided by the Z-test for uncertainty events under classical statistics is 0.94 and the power of the test provided by the proposed Z-test for uncertainty events is 0.96. The values of the power of the test for Z-test for uncertainty events under classical statistics and Z-test for uncertainty events under neutrosophic statistics are plotted in Fig. 1. From Fig. 1, it is quite clear that the power curve of the proposed test is higher than the power curve of the existing test. Based on the analysis, it can be concluded that the proposed Z-test for uncertainty events under neutrosophic statistics is efficient than the existing Z-test for uncertainty events.

**Table 2:** The values of the power of two tests

${I}_{\mu_U}$	Existing Test	The Proposed Test
	1 − *β*	1 − *β*
0.05	0.96	0.97
0.1	0.94	0.96
0.2	0.94	0.96
0.3	0.91	0.97
0.4	0.93	0.95
0.5	0.89	0.95
0.75	0.89	0.92
1	0.93	0.96

## Conclusions

The Z-test of uncertainty was introduced under neutrosophic statistics in this paper. The proposed test was a generalization of the existing Z-test of uncertain events under classical statistics, fuzzy-based test, and interval statistics. The performance of the proposed test was compared with the listed existing tests. From the real data and simulation study, the proposed test was found to be more efficient in terms of information and power of the test. Based on the information, it is recommended to apply the proposed test to check the reduction in uncertainty under an indeterminate environment. The proposed test for big data can be considered as future research. The proposed test using double sampling can also be studied as future research. The estimation of sample size and other properties of the proposed test can be studied in future research.

## Data Availability

All data generated or analysed during this study are included in this published article
